# Pregnancy, parturition and preeclampsia in women of African ancestry

**DOI:** 10.1016/j.ajog.2013.10.879

**Published:** 2014-06

**Authors:** Annettee Nakimuli, Olympe Chazara, Josaphat Byamugisha, Alison M. Elliott, Pontiano Kaleebu, Florence Mirembe, Ashley Moffett

**Affiliations:** a Department of Obstetrics and Gynaecology, Makerere University and Mulago Hospital, Kampala, Uganda; b Department of Pathology and Centre for Trophoblast Research, University of Cambridge, Cambridge, United Kingdom; c Medical Research Council/Uganda Virus Research Institute Uganda Research Unit on AIDS, Entebbe, Uganda; d London School of Hygiene and Tropical Medicine, London, United Kingdom

**Keywords:** evolutionary selective pressure, great obstetric syndromes, length of gestation, obstetric dilemma

## Abstract

Maternal and associated neonatal mortality rates in sub-Saharan Africa remain unacceptably high. In Mulago Hospital (Kampala, Uganda), 2 major causes of maternal death are preeclampsia and obstructed labor and their complications, conditions occurring at the extremes of the birthweight spectrum, a situation encapsulated as the obstetric dilemma. We have questioned whether the prevalence of these disorders occurs more frequently in indigenous African women and those with African ancestry elsewhere in the world by reviewing available literature. We conclude that these women are at greater risk of preeclampsia than other racial groups. At least part of this susceptibility seems independent of socioeconomic status and likely is due to biological or genetic factors. Evidence for a genetic contribution to preeclampsia is discussed. We go on to propose that the obstetric dilemma in humans is responsible for this situation and discuss how parturition and birthweight are subject to stabilizing selection. Other data we present also suggest that there are particularly strong evolutionary selective pressures operating during pregnancy and delivery in Africans. There is much greater genetic diversity and less linkage disequilibrium in Africa, and the genes responsible for regulating birthweight and placentation may therefore be easier to define than in non-African cohorts. Inclusion of African women into research on preeclampsia is an essential component in tackling this major disparity of maternal health.

More than 90% of maternal deaths worldwide occur in sub-Saharan Africa (SSA) and south Asia. These high maternal and associated neonatal mortality rates persist despite considerable efforts from the World Health Organization, governments, development partners, and others.^1^, ^2^, ^3^ The majority of these deaths are related to pregnancy complications that are inadequately managed because of a lack of access to emergency health care. The maternal mortality ratios (MMRs) of Sweden, the United Kingdom, and the United States are 4, 12, and 21, respectively, whereas those of Chad, Nigeria, and Congo are 1100, 630, and 540 per 100,000 live births, respectively. In SSA, the major direct causes of maternal mortality are hemorrhage, preeclampsia/eclampsia, obstructed labor, and sepsis.^4^ Infections, preterm birth, birth asphyxia, stillbirths, and small-for-gestational-age infants are the leading causes of perinatal mortality.^2^, ^5^

These observations are representative of our own institution, Mulago Hospital in Kampala (Uganda) in which the MMR has remained high at 438 per 100,000 live births, even though there has been an increase in skilled birth attendance (58%) and very good attendance rate (95%) at antenatal clinics.^6^

Mulago Hospital is the busiest maternity hospital in SSA, serving as a tertiary referral center for Uganda. Details of deliveries and maternal deaths are shown in Table 1. Even with the lack of good medical records that is characteristic of much of SSA, our experience in Mulago Hospital is that causes of maternal deaths are similar to the rest of SSA, with hemorrhage, preeclampsia/eclampsia, and sepsis occurring very commonly.^4^, ^7^, ^8^ The large number of women seen with preeclampsia, particularly recurrent, severe, and early-onset preeclampsia and eclampsia, is of particular concern to us because these conditions have a high mortality and morbidity, are impossible to predict, and their pathogenesis is still somewhat mysterious.

**Table 1:** Data for the maternity unit in Mulago Hospital, Uganda

Variable	2009	2010	2011
Live births	30,247	31,585	32,633
Stillbirths	1260	1303	1230
Cesarean sections	6849	6702	6800
Ruptured uterus	125	119	206
Maternal deaths	187	152	188
Attendance at antenatal clinic	78,157	76,673	69,129

*Nakimuli. Pregnancy, parturition and preeclampsia in Africa. Am J Obstet Gynecol 2014*.

Here we review data relating to preeclampsia in indigenous Africans and in women of African ancestry elsewhere in the world. We discuss the idea that in these women, apart from the obvious, cultural and socioeconomic factors and different priorities in health care, there are additional biological reasons why the preeclampsia syndromes are such a prominent feature of African obstetrics. Our findings also lead us to question whether there are other characteristics of pregnancy and parturition that differ in African women.

## Preeclampsia and the great obstetric syndromes

An important determinant of preeclampsia is failure of placentation, particularly the physiological transformation of spiral arteries, which leads to a stressed, underperfused placenta.^9^, ^10^ Preeclampsia is one of a spectrum of pregnancy disorders that may result from this underlying pathogenesis, including fetal growth restriction (FGR), stillbirth, abruptio placentae, and some cases of preterm labor with intact membranes and prelabor rupture of membranes.^11^, ^12^ Because of the overlap in these conditions, it is useful to think of them together as the great obstetric syndromes (GOS) (Appendix).^13^, ^14^, ^15^

All these conditions are seen very frequently in Mulago Hospital. However, FGR cannot be reliably diagnosed without accurate knowledge of gestational age, and low birthweight may result from a variety of causes. Similarly, stillbirth is a heterogeneous condition that can result from congenital infection, birth asphyxia, or birth trauma as well as poor uteroplacental perfusion.

Because preeclampsia is a recognized clinical entity characterized by new onset of hypertension and proteinuria after 20 weeks' gestation, we have focused on this disorder.^16^, ^17^ The exact prevalence of preeclampsia in SSA is unknown because detailed clinical records of all births are lacking. Distinguishing between true preeclampsia and pregnancy-induced hypertension is also difficult because proteinuria may not be adequately measured. A further problem is a lack of information on preexisting hypertension because presentation to the clinic is often late.

Given this dearth of accurate records of pregnancy outcomes in Uganda and SSA generally, to review the incidence of preeclampsia in women of African ancestry, we have reviewed reports relating to preeclampsia in African Americans (AA) and immigrants from Africa to other high-income countries as compared with other ethnic groups. Studies were identified through a search of the PubMed database for relevant peer-reviewed articles published in English using the search terms, preeclampsia or eclampsia or hypertensive disease in pregnancy or gestational hypertension or severe maternal morbidity and ethnicity or race (Tables 2 and 3).

**Table 2:** Preeclampsia or eclampsia studies among African Americans

Cohort size (total/AA)	Preeclampsia or eclampsia, OR (95% CI)	Comments	Reference
2,571,069/450,098	1.67 (1.64–1.71)^a^	PE in women in New York state	^23^
1,030,350/161,780	1.59 (1.49–1.69)	Adjusted for maternal characteristics and obstetric history	^24^
1,472,912/420,576	1.30 (1.28–1.33)	Adjusted for maternal characteristics and obstetric history	^25^
299,499/n.a.	1.39 (1.26–1.54)^a^	Severe PE in women without chronic hypertension	^26^
206,428/19,512	2.12 (1.85–2.42)	Adjusted for maternal characteristics and obstetric history	^27^
330/124	2.25 (0.88–5.78)	Eclampsia, adjusted for maternal characteristics and obstetric history	^29^
4702/740	1.40 (1.20–1.80)	Adjusted for maternal characteristics and obstetric history	^30^
271/38	2.50 (0.97–6.40)	Adjusted for maternal characteristics and obstetric history	^31^
4314/1998	1.23 (0.88–1.72)	Adjusted for maternal characteristics and obstetric history	^32^
153/35	2.27 (1.26–5.92)	Late postpartum PE, not adjusted	^33^
2394/592	1.53 (1.00–2.35)	Adjusted for maternal characteristics, obstetric history, and biochemical factors	^34^
103,860/13,748	1.36 (1.27–1.45)^a^	PE in women with singleton birth at first delivery	^35^
2,770,871/121,017	1.81 (1.51–2.17)	Eclampsia, adjusted for maternal characteristics and obstetric history	^36^
127,544/12,639	1.41 (1.25–1.62)	Adjusted for maternal characteristics, chronic hypertension excluded	^28^
16,300/6000	1.63 (1.58–1.69)^a^	Eclampsia in racial minorities, not adjusted, not significant	^38^
1355/374	3.20 (1.04–9.93)	PE in women without chronic hypertension	^40^
500/68	2.29 (1.16–4.53)	Recurrent PE, adjusted for maternal characteristics and obstetric history	^67^
10,755/5555	1.30 (1.07–1.58)	Adjusted for maternal characteristics	^44^
2947/156	1.62 (0.00–3.20)	No effect when analyzed by recruitment center	^69^

Maternal characteristics generally include maternal age, body mass index, and smoking. Obstetric history generally includes parity, chronic hypertension, and diabetes.

*CI*, confidence interval; *n.a.*, not available; *OR*, odds ratio; *PE*, preeclampsia.

*Nakimuli. Pregnancy, parturition and preeclampsia in Africa. Am J Obstet Gynecol 2014*.

a OR was calculated from the data.

**Table 3:** Preeclampsia studies among recent African immigrants to other countries

Cohort size (total/Africans)	Preeclampsia, OR or RR (95% CI)	African origin	Comments	Location	Reference
118,849/15,218	3.34 (2.25–4.96)	Caribbean	Adjusted for maternal characteristics	Canada	^49^
118,849/9130	3.14 (2.04–4.83)	SSA	Adjusted for maternal characteristics	Canada	^49^
2413/317	2.40 (1.10–5.60)	SSA, Surinam, Antilles	Univariate analysis	The Netherlands	^50^
2506/29	2.70 (1.20–6.20)	Antilles	Eclampsia in cases of SAMM, adjusted for maternal characteristics and obstetric history	The Netherlands	^51^
2506/90	6.20 (3.60–10.6)	SSA	Eclampsia in cases of SAMM, adjusted for maternal characteristics and obstetric history	The Netherlands	^51^
6215/331	2.06 (1.04–4.09)	Cape Verde	Adjusted for maternal characteristics and obstetric history	The Netherlands	^52^
6215/264	1.87 (0.86–4.06)	Antilles	Adjusted for maternal characteristics and obstetric history	The Netherlands	^52^
1728/576	2.47 (1.02–6.00)	Ethiopia	Standardized care between the groups compared	Israel	^53^
76,158/11,395	2.60 (2.32–2.92)	Caribbean	Adjusted for maternal characteristics and obstetric history	United Kingdom	^54^
8366/1581	3.64 (1.84–7.21)	n.a.	Adjusted for maternal characteristics and obstetric history	United Kingdom	^55^
15,639/356	0.90 (0.53–1.51)^a^	SSA	No increased risk	Sweden	^56^
165,001/986	n.a.	African, Somalia	No increased risk	Finland	^58^
526/158	3.90 (1.70–8.94)^a^	SSA	Early-onset PE compared to late onset (<28 or <34 weeks)	France	^68^

Maternal characteristics generally include maternal age, body mass index, and socioeconomic status. Obstetric history generally includes parity, chronic hypertension, and diabetes.

*CI*, confidence interval; *n.a.*, not available; *OR*, odds ratio; *PE*, preeclampsia; *RR*, relative risk; *SAMM*, severe acute maternal morbidity.

*Nakimuli. Pregnancy, parturition and preeclampsia in Africa. Am J Obstet Gynecol 2014*.

a OR was calculated from the data.

In this review, we designate women of African ancestry as those women descended from inhabitants of SSA. There are obvious caveats when reviewing data from women of African descent who have migrated to new environments. Those who have the energy to migrate may be healthier than those left behind. Furthermore, factors such as diet, lifestyle, education, health care, climate, and indigenous pathogens are different and necessarily become an integral part of the immigrant's new environment.

## Preeclampsia among African Americans

Although African Americans are obviously not directly comparable with indigenous Africans because of considerable genetic admixture (7-23%),^18^, ^19^ the large number of reports and the consistency of the findings are informative (Table 2). For decades it has been clear that there are disparities in obstetric outcomes including preeclampsia between AA and other groups; indeed, black ethnicity is cited as a risk factor for preeclampsia in reviews.^20^, ^21^ Of 4 million births recorded in the National Vital Statistics Report, pregnancy-associated hypertension was more common in AA (5.0%) and least frequent in Hispanics (2.9%).^22^

A study of more than 2 million pregnancies in New York using data from hospital discharge records found that the rates of preeclampsia were substantially higher among AA compared with European Americans. This was even more obvious when confounders such as diabetes and maternal age were taken into account. Furthermore, the difference persisted after stratification for socioeconomic status based on area of residence.^23^

Two other large studies in the United States, each with more than 1 million women also found that preeclampsia was more common in AA compared with European Americans.^24^, ^25^ One of these studies took data from the National Inpatient Sample in which information was also available on health insurance and income level; when this was taken into account, the findings remained the same.^24^ The other study used data from women who were all Medicaid enrollees in 14 southern states.^25^ AA women were also most likely to have other poor maternal outcomes like preterm labor, abruptio placenta, and stillbirth.

Another large study from the National Hospital Discharge Survey found AA women had a higher incidence of all hypertensive disorders in pregnancy and a greater risk of severe complications of preeclampsia such as abruptio placenta and stillbirth compared with European Americans.^26^ Similar findings were made in a large Wisconsin study recruited from hospital discharge data. AA women had the highest risk for all the different types of preeclampsia when compared with European American women.^27^ Several other studies looking at risk factors for both eclampsia and preeclampsia in nulliparous and parous women have also shown AA are at higher risk (Table 2).^28^, ^29^, ^30^, ^31^, ^32^, ^33^, ^34^, ^35^, ^36^

Confounding factors such as obesity, preexisting chronic hypertension, and diabetes are difficult to control for and are likely to contribute to the increased risk of preeclampsia among AA, particularly in the case of chronic hypertension.^37^, ^38^, ^39^ That preeclampsia may not be wholly explained by higher rates of chronic hypertension among AA women is suggested by a comparison between African and European Americans without chronic hypertension; the prevalence of hypertension in pregnancy was similar, but AA women still had an increased diagnosis of preeclampsia.^40^ Similar findings were made much earlier by the Collaborative Perinatal project, which revealed a higher incidence of preeclampsia and eclampsia among AA women compared with their European counterparts, irrespective of whether there was preexisting hypertension.^41^

Investigation of the GOS other than preeclampsia is more difficult because of the problems in accurate diagnosis described above. Nonetheless, a consistent message is that ethnic disparities exist for all the GOS (spontaneous preterm labor, FGR, stillbirth, and other poor obstetric outcomes), and all have an increased frequency among AA.^26^, ^42^, ^43^, ^44^, ^45^ In a study of more than 5 million births comparing birth outcomes between US-born and foreign-born women, women of African ancestry had the highest rates of infant mortality, low birthweight, and preterm births, whether US born or foreign born.^46^ In addition, the risk of preterm birth, stillbirth, and low birthweight is increased not only in AA women but also with AA fathers.^47^, ^48^

Explanations for the disparities found between women with African or European ancestry have been poor socioeconomic status with lower incomes and level of education, lack of medical insurance, poor utilization of preconception and antenatal services, stress, discrimination, and residential segregation. Several reports have tried to determine the impact of these factors; for example, women of African ancestry were at an increased risk of preeclampsia in a second pregnancy, but this was not associated with Medicaid enrollment.^30^

Many of the socioeconomic factors that may contribute to poor obstetric outcomes also apply to the Hispanic population in the United States, yet several studies have noted that preeclampsia, low birthweight, and stillbirth are similar or even better than for white women, the Hispanic paradox.^28^, ^44^ Using information from the Duke University Birth Database, AA women had higher rates of preeclampsia (10.2%) than the European (8%) or Hispanic women (6.2%), even though the socioeconomic status of Hispanic and AA women was similar.^44^

## Preeclampsia among more recent African immigrants to other countries

Large numbers of Africans have migrated to Europe and other high-income countries, mainly in the past 50 years. Obstetric outcomes for these recent African immigrants are informative, particularly because these births often take place in countries with good records and universal health care systems (Table 3). For example, a large study of more than 100,000 women who immigrated to Ontario between 1985 and 2000 showed that the racial groups with the highest risk of severe preeclampsia were from the Caribbean or SSA.^49^

Similar findings were made in The Netherlands where the highest risk for eclampsia and preeclampsia was from women from SSA.^50^, ^51^ Cape Verdean and Antillean women were also at higher risk of preeclampsia in a report from Rotterdam, The Netherlands.^52^ A large number of Ethiopians have settled in Israel since the 1980s where prenatal and obstetric care is standardized with equal medical insurance, and in this group severe preeclampsia was more likely to occur.^53^

Large groups of women of African ancestry live in London where access to National Health Service hospitals is freely available and home deliveries are rare. In a survey of 80,000 pregnancies that included women of European and Asian ancestry, 15% had African ancestry, and this was the second strongest risk factor for preeclampsia after chronic hypertension and also carried a higher risk of other poor obstetric outcomes such as FGR and stillbirth.^54^ Similarly, African ancestry was a risk factor for early-onset preeclampsia compared with all other racial groups, and this remained so, even after adjusting for age, body mass index, and other maternal characteristics.^55^ These and other studies of African immigrants also highlight the increased risk of GOS such as stillbirths and FGR similar to AA.^56^, ^57^, ^58^

## Severity and recurrence of preeclampsia

The early onset and severity of preeclampsia in women from Uganda is also a cause of concern, although the latter may reflect the late admittance to Mulago Hospital. In a US national hospital discharge survey, higher mortality from preeclampsia and eclampsia was reported among women of African ancestry compared with European Americans, but only one-third or less of the difference could actually be attributed to the higher prevalence.^59^ Pregnancy-related deaths from preeclampsia/eclampsia were 3 times higher in AA women compared with Europeans.^60^

In the UK Maternal Death Review for the period 2006-2008, 22 deaths occurred as a result of preeclampsia and eclampsia. Despite being a minority group, 6 of these deaths were Africans and the authors noted: “Black African women seem particularly susceptible to aggressive forms of preeclampsia. To establish if this is true, and what might be the underlying genetic or other pathophysiological mechanisms, further research is required.”^61^

After a woman has had preeclampsia in her first pregnancy, the risk of recurrence is increased, with a relative risk of 15.0 cited in an authoritative Norwegian study of more than 2 million women.^62^ Increased risk of other GOS, even if preeclampsia does not occur, is also clear from another large study in Sweden, and other reports support this conclusion.^35^, ^63^, ^64^, ^65^, ^66^

Large studies of this kind are still not available for African women resident in SSA, but our own experience in Kampala is that recurrent preeclampsia does occur frequently. In London, 23% of 500 women with previous preeclampsia had recurrent disease that required delivery before 37 weeks, and African compared with European ancestry was a significant predictor.^67^ It also seems that when preeclampsia does occur in the second pregnancy in AA women, it is severe, early-onset disease with associated FGR and preterm birth.^45^ A recent study from France suggests that women of African ancestry are more at risk for early-onset preeclampsia and more likely to have had a previous history of preeclampsia compared with other groups, including women from North Africa, despite the even higher incidence of chronic hypertension in the latter group.^68^

## Summary

Our comprehensive review of the literature identified very few papers that run counter to our conclusion that women of African ancestry are at increased risk of developing preeclampsia. First, 3 studies showed that these women were not at increased risk of preeclampsia, but they had low power to detect any effect.^56^, ^58^, ^69^ Second, the apparent increased susceptibility to preeclampsia among AA has been dismissed as a problem of incorrect diagnosis.^37^ Third, race was discounted as a significant risk factor for preeclampsia in another study, but data regarding AA women were combined with that for other minority races so that the analysis could not provide a meaningful comparison.^38^

## Genetics of preeclampsia

That there is a genetic component to preeclampsia has long been suspected.^70^, ^71^, ^72^ Daughters of women with preeclampsia have more than twice the risk of developing the disease themselves, and sisters of affected women, even if not born from a preeclamptic pregnancy, are also at increased risk.^73^, ^74^, ^75^, ^76^, ^77^ These findings of familial aggregation in preeclampsia are also true for the other GOS.^78^, ^79^, ^80^, ^81^ Although environmental factors, particularly influences acting in utero, are important, some of the risk is likely to be genetic. Indeed, a study of female twin pairs with known zygosity estimated that the heritability of preeclampsia was approximately 54%.^82^ Could there be particular susceptibility genes associated with the higher frequency of preeclampsia in women of African ancestry? A case-control study of preeclampsia in Latinas, a group with admixture from European, African, and native Americans, did show, using ancestry informative markers, that African ancestry was associated with preeclampsia.^83^

The role of the fetal (father's) genes is less obvious, but many reports indicate a paternal contribution to the risk.^84^, ^85^, ^86^, ^87^ Intergenerational and familial aggregation also point to genetic factors derived from both maternal and fetal genes, with most risk coming from maternal genes that may act in either the mother or her fetus.^71^, ^74^, ^77^, ^88^ A drive to look for the susceptibility genes for preeclampsia has so far been disappointing. The studies generally have small numbers of subjects and have not been replicated.^89^

Genome-wide association screening is an unbiased approach to look for susceptibility genes in complex disorders and has been used in preeclamptic cohorts, but, although various single-nucleotide polymorphism candidates have been identified, the lack of statistical power is again a problem.^90^, ^91^, ^92^, ^93^ Systematic metaanalyses of these studies found 7 single-nucleotide polymorphisms significantly associated near genes involved in processes such as coagulation, the renin-angiotensin system, and inflammation.

This highlights an important issue: searching for variants associated with preeclampsia only in the maternal genome will reveal genes mainly associated with the tertiary systemic syndrome and not those maternal and/or fetal genes involved in physiological transformation of the arteries or to the subsequent stress response of the placenta to the reduced blood flow. The clear increased risk of cardiovascular disease in women who have had preeclampsia again points to a separate set of susceptibility genes that are acting systemically and not during early placentation.^94^, ^95^

We have taken a different approach and focused on the primary defect of poor placentation. This is based on the idea that regulation of trophoblast behavior during placentation is mediated by allogeneic recognition of trophoblast major histocompatibility complex molecules by maternal lymphocytes.^96^ The findings that specialized immune cells, uterine natural killer (NK) cells, accumulate at the site of placentation, together with the discovery of NK receptors, the killer-cell immunoglobulin-like receptor family (KIR) and their cognate HLA-C trophoblast ligands have demonstrated how the mother can discern the presence of a genetically different individual.^97^, ^98^, ^99^

*KIR* and *HLA* are the most polymorphic gene families in humans, and we have shown that particular maternal *KIR* in combination with fetal *HLA-C* variants are associated with preeclampsia and the other GOS.^100^, ^101^, ^102^ Women who have 2 *KIR A* haplotypes (*KIR AA* genotype) are at risk when there is a *HLA-C* allele belonging to the *C2* group in the fetus. Furthermore, the origin of the fetal *HLA-C2* is important; the most risk is from a *C2* allele inherited from the father.

We are now undertaking a similar study at Mulago Hospital, and preliminary findings illustrate the same maternal *KIR*/fetal *HLA-C* combinations associated with preeclampsia in African women. Interestingly, the frequency of the fetal *HLA-C2* variant that confers risk is increased in Ugandans compared with Europeans and Asians.^103^ Furthermore, there is enormous variability of *KIR* genes in Africans with far more genotypes and more allelic variation at individual *KIR* loci.^104^

**Figure 1:**
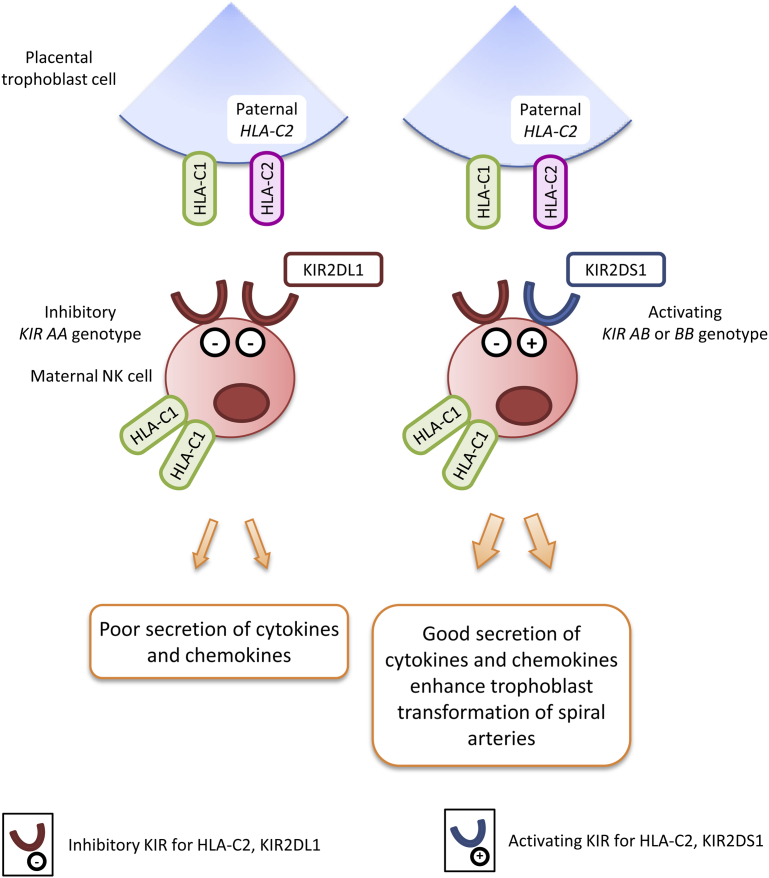
Maternal KIR/fetal HLA-C interactions at the site of placentation In these 2 scenarios, the mother is *HLA-C1* homozygous and the fetus has inherited an *HLA-C2* group allele from the father. If the mother has a *KIR AA* genotype that lacks activating KIR and has a strong inhibitory KIR for HLA-C2 (KIR2DL1), poor placentation results. In contrast, if the mother has a *KIR AB* or *BB* genotype containing the activating KIR for HLA-C2 (KIR2DS1), uterine natural killer cells are triggered to produce increased amount of cytokines and chemokines (eg, granulocyte-macrophage colony–stimulating factor) that enhance placentation. *Nakimuli. Pregnancy, parturition and preeclampsia in Africa. Am J Obstet Gynecol 2014*.

How these genetic findings translate into the function of uterine NK cells is a challenge, given the ethical and logistical difficulties in experimenting with these cells. Functionally, we would predict that the risky combination results in very strong inhibition of uterine NK cells (Figure 1). Triggering of uterine NK cells by HLA-C2 target cells in vitro from women who have a protective *KIR B* haplotype (in which the activating *KIR* for HLA-C2, *KIR2DS1*, is located) results in secretion of cytokines and chemokines that may facilitate trophoblast invasion and vascular transformation.^105^ Thus, we propose that the uterine immune system using highly variable maternal KIR/fetal HLA-C interactions subtly defines the boundary between mother and baby, limiting the highly invasive placenta while at the same time ensuring the fetus receives sufficient nourishment for normal development through remodeling of the spiral arteries.

## The obstetric dilemma

Overall, the data we have brought together in this review suggest that preeclampsia and other GOS occur more commonly in women of African ancestry compared with other ethnic groups, and this is not wholly accounted for by confounding social, cultural, and medical influences.^50^, ^54^, ^57^, ^68^ It also seems that the risk of preeclampsia in African immigrants to Europe is increased irrespective of their area of origin in Africa, apart from North Africans.^68^ All these observations point to a need to investigate possible biological/genetic reasons contributing to the higher risk of preeclampsia in SSA.

We would anticipate that there would be strong selective pressure against a disorder that, without medical intervention, is frequently fatal to mother and child and occurs in 5-10% of first pregnancies. A failure of the physiological transformation of uterine arteries is a common feature of all the GOS, and this results in a reduced placental supply of oxygen and nutrients, lower birthweights, and the risk of preterm labor and superimposed preeclampsia. However, at the same time, we have to consider that maternal and neonatal mortality is not only high under circumstances of reduced fetal nutrition but also when babies are too large for the pelvis.

**Figure 2:**
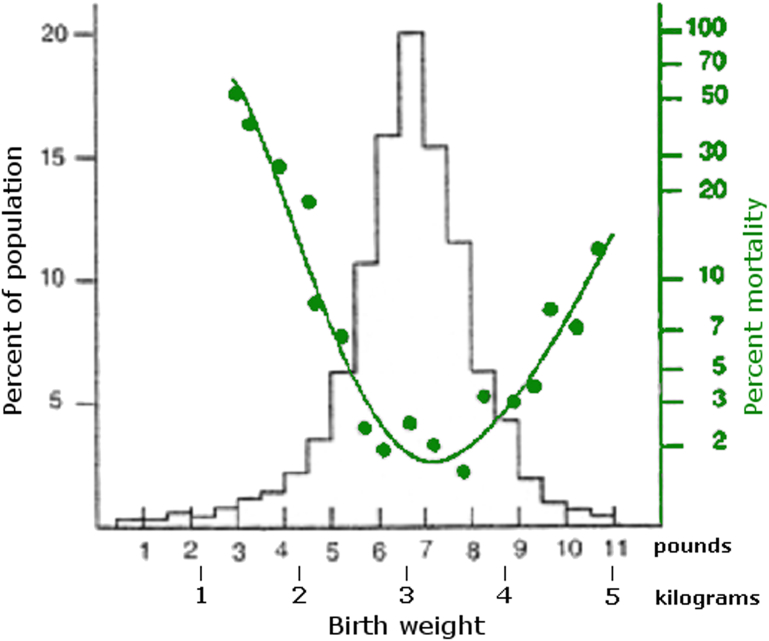
Birthweight and neonatal mortality rates (n = 13,730). *Nakimuli. Pregnancy, parturition and preeclampsia in Africa. Am J Obstet Gynecol 2014.*

Compared with other primates, the passage of the large human fetal head through a bony pelvis is a tight fit, requiring rotation of the head as it goes through the birth canal as a consequence of adaptation to bipedalism.^106^, ^107^ The high maternal and neonatal mortality associated with extremes of birthweight, sometimes called the obstetric dilemma, has been described as “perhaps the most clear-cut example of a human character subject to stabilizing selection” (Figure 2).^108^

Adapted, with permission, from Cavalli-Sforza and Bodmer.^108^

The optimal survival of babies weighing between 6 and 8 lb (2.5-3.5 kg) seems to be a universal feature of human populations. If babies become too large, the risk of obstructed labor is increased. As in the rest of SSA, at Mulago Hospital we not only have many disordered pregnancies arising from failure of placentation, but we also experience frequent births with prolonged obstructed labor because of cephalopelvic disproportion. Without cesarean section, this leads to birth asphyxia, postpartum hemorrhage, pelvic trauma, sepsis, and long-term problems such as vesicovaginal fistula.^109^ In Uganda, 2% of all women have had an obstetric fistula.^6^ Therefore, the higher cesarean section rates seen in high-income countries in women of African ancestry may reflect not just delivery of women with preeclampsia but also an increased frequency of obstructed labor.^24^, ^54^, ^58^, ^110^

A detailed audit from the Royal College of Obstetricians and Gynaecologists in the United Kingdom highlighted the higher cesarean section rates in women of African ancestry, even when confounders such as age, parity, birthweight, and presentation were considered.^110^ Furthermore, shoulder dystocia has also been reported to occur more commonly in AA women.^111^

These findings may in part be accounted for by measurements of the bony pelvis revealing that there is even less room for the fetal head in women of African ancestry. Although pelvimetry may not be a useful indicator in predicting cephalopelvic disproportion in individual patients,^112^ the measurements made of the pelvis, notably the pelvic inlet, outlet, length of sacrum, and pelvic floor area, are all smaller in women of African ancestry compared with those of European ancestry.^113^, ^114^, ^115^

A possible consequence is that the fetal head engages into the pelvis late, only when labor commences, whereas this occurs in the last month of gestation in European and Asian women.^116^ Several reports also document the fact that normal term in African pregnancy occurs at only approximately 38 weeks' gestation, which is 2 weeks earlier than in non-Africans, possibly facilitating birth before the baby becomes too big.^117^, ^118^, ^119^, ^120^ If births are occurring earlier, it would certainly then be advantageous for the fetus to mature more quickly, and indeed, African babies frequently pass meconium with no sign of fetal distress.^119^, ^121^ There is also accelerated lung maturity.^122^, ^123^ Thus, the difficulties imposed by the birth process and the passage of the head and shoulders through the bony pelvis seem to have driven biological changes in many aspects of pregnancy.

This clear trade-off between the size of the pelvis and head room will benefit babies who have intermediate birthweights, and the 2 extremes with reduced survival will be selected against. Of interest will be a study of maternal *KIR*/fetal *HLA-C* variants in pregnancies with obstructed labor as the *KIR A* and *B* haplotypes and *HLA-C1* and *C2* groups are under stabilizing selection and are found at different frequencies across the world's populations.^124^

Therefore, when considering why preeclampsia persists in populations, it is important not only to consider the GOS but also the morbidity and mortality at the other end of the birth weight distribution. It may be that the consequences of these contrasting selective pressures not only affect higher birthweight babies but also the tendency for enhanced numbers of undernourished babies with the concomitant maternal syndrome of preeclampsia. That is to say that selection in a population for reduced fetal size may lead to the persistence of factors predisposing to preeclampsia.

## Future directions

There is an urgent need to document obstetric events better in SSA, and the lack of detailed electronic hospital records in the hospitals as well as the failure to record all births in the population is a major difficulty for any maternal health research program. For example, proper assessment of gestational age at delivery will be crucial in the accurate diagnosis of the GOS. The record systems documenting obstetric and neonatal problems are still inadequate throughout SSA, and this is clearly a major priority. Introduction of record systems in Zambia had an immediate impact on health care such as penicillin administration for syphilis as well as highlighting the shorter gestational age and describing the normal birthweight distribution.^125^

The majority of studies on preeclampsia and the other GOS are from Europe and the Americas, but if these conditions are indeed occurring at greater frequency in women of African ancestry, it makes scientific and economic sense to study them in the setting in which they have a major impact. Furthermore, the out-of-Africa migrations have reduced the extent of genetic variation in the European populations that are the focus of the great majority of studies of pregnancy disorders.

Studying biological diversity will shed light on pathological pregnancies in all populations, and inclusion of African women into research on preeclampsia is an essential component in tackling this major disparity of maternal health. It has been highlighted that “Africa is a genetically special place” with greater genetic diversity and lower levels of linkage disequilibrium.^126^ The genes contributing to defective placentation are therefore likely to become obvious more quickly. Indeed, our initial study of *KIR* genes has found a much greater number of different *KIR* haplotypes in women delivering at Mulago Hospital but also highlighted the extent of variability of these genes within the African continent.^103^ Denying the existence of genetic differences in Africans and their interactions with nongenetic factors only delays the identification of the causal genes or alleles that would allow us to move away from racial/ethnic categorization of individuals. Knowing the genetic variants will allow a better understanding of the molecular pathways and better health care for the women carrying the risky genotypes, independently from their ethnicity.

## Appendix

### Glossary

**Genetic admixture:** phenomenon of interbreeding between members of 2 or more different populations resulting in the exchange of genes.

**Genome-wide association:** a search for common genetic variants associated with a disease. Generally, the association of particular single-nucleotide polymorphism (SNPs), variation in the DNA sequence of a single nucleotide, is analyzed in individuals with the disease compared with normal matched control subjects.

**Great obstetrical syndromes:** major pregnancy complications such as spontaneous abortion, fetal death, abruptio placentae, fetal growth failure, preeclampsia, preterm labor, and premature rupture of membranes. These disorders all share the primary defect of failure of physiological transformation of uterine spiral arteries, but there are multiple fetal and maternal factors that determine the exact clinical outcome.

**HLA-C:** polymorphic classical HLA class I molecules present at the surface of most nucleated cells and also expressed by invasive trophoblast cells at the site of placentation. HLA-C molecules are important ligands for KIR receptors.

**Killer cell immunoglobulin-like receptors (KIR):** a family of closely related genes located in the leukocyte receptor complex on chromosome 19. KIR proteins are expressed on the surface of NK cells. KIR can have an activating or inhibitory effect on NK cell function. In all populations, 2 types of KIR haplotypes are found; KIR A haplotypes contain mainly inhibitory KIR, whereas KIR B haplotypes contain extra, mainly activating, KIR.

**Linkage disequilibrium:** differences in a population between the observed occurrence of some combinations of alleles or genetic markers and their expected frequencies under the assumption of independence. Linkage disequilibrium is expected to be the greatest for 2 genes located closely together on a chromosome.

**NK cells:** large granular lymphocytes of the innate immune system that play important roles in defense against viral infections and tumors. A specialized subset of NK cells amass at the site of placentation early in gestation.

**Positive selection:** type of natural selection that results in the increase of the frequency of the advantageous alleles over time.

**Stabilizing selection:** selection favoring intermediate values of a character over the extremes.
